# Induction of Inducible Nitric Oxide Synthase by Lipopolysaccharide and the Influences of Cell Volume Changes, Stress Hormones and Oxidative Stress on Nitric Oxide Efflux from the Perfused Liver of Air-Breathing Catfish, *Heteropneustes fossilis*

**DOI:** 10.1371/journal.pone.0150469

**Published:** 2016-03-07

**Authors:** Mahua G. Choudhury, Nirmalendu Saha

**Affiliations:** Biochemical Adaptation Laboratory, Department of Zoology, North-Eastern Hill University, Shillong, 793 022, India; University of Louisville, UNITED STATES

## Abstract

The air-breathing singhi catfish (*Heteropneustes fossilis*) is frequently being challenged by bacterial contaminants, and different environmental insults like osmotic, hyper-ammonia, dehydration and oxidative stresses in its natural habitats throughout the year. The main objectives of the present investigation were to determine (a) the possible induction of inducible nitric oxide synthase (iNOS) gene with enhanced production of nitric oxide (NO) by intra-peritoneal injection of lipopolysaccharide (LPS) (a bacterial endotoxin), and (b) to determine the effects of hepatic cell volume changes due to anisotonicity or by infusion of certain metabolites, stress hormones and by induction of oxidative stress on production of NO from the iNOS-induced perfused liver of singhi catfish. Intra-peritoneal injection of LPS led to induction of iNOS gene and localized tissue specific expression of iNOS enzyme with more production and accumulation of NO in different tissues of singhi catfish. Further, changes of hydration status/cell volume, caused either by anisotonicity or by infusion of certain metabolites such as glutamine plus glycine and adenosine, affected the NO production from the perfused liver of iNOS-induced singhi catfish. In general, increase of hydration status/cell swelling due to hypotonicity caused decrease, and decrease of hydration status/cell shrinkage due to hypertonicity caused increase of NO efflux from the perfused liver, thus suggesting that changes in hydration status/cell volume of hepatic cells serve as a potent modulator for regulating the NO production. Significant increase of NO efflux from the perfused liver was also observed while infusing the liver with stress hormones like epinephrine and norepinephrine, accompanied with decrease of hydration status/cell volume of hepatic cells. Further, oxidative stress, caused due to infusion of t-butyl hydroperoxide and hydrogen peroxide separately, in the perfused liver of singhi catfish, resulted in significant increase of NO efflux accompanied with decrease of hydration status/cell volume of hepatic cells. However, the reasons for these cell volume-sensitive changes of NO efflux from the liver of singhi catfish are not fully understood with the available data. Nonetheless, enhanced or decreased production of NO from the perfused liver under osmotic stress, in presence of stress hormones and oxidative stress reflected its potential role in cellular homeostasis and also for better adaptations under environmental challenges. This is the first report of osmosensitive and oxidative stress-induced changes of NO production and efflux from the liver of any teleosts. Further, the level of expression of iNOS in this singhi catfish could also serve as an important indicator to determine the pathological status of the external environment.

## Introduction

Nitric oxide (NO), one of the smallest known bioactive products of cells, is known to act as an intra- and extracellular mediator of various cell functions [1,2]. NO produced by the constitutive isoform of nitric oxide synthase (NOS) is well documented to be involved in homeostasis, whereas NO which is produced by inducible NOS (iNOS) plays a major role in neurotransmission, cytotoxicity, cytoprotection, inflammation, host-defense responses, protect against oxidative and hypoxia stress [3–7].

The productions of NO during different stresses and its role in different stress-related reactions have been well documented mostly in mammalian system. Evaluating the effects of various stressors on NO production, different authors have found both increases and decreases in NO production, and suggested that the increased NO production corresponds to the stage of mobilization in response to the appropriate stress reaction, while the decreased NO production corresponds to the stage of exhaustion in excess stress reaction [8]. The stress-induced increase in NO synthesis can occur both due to activation of pre-existing NOS [9,10] and also at the expense of *de novo* increased formation of NOS [11].

A remarkable property of living cells is their ability to maintain a comparatively constant cell volume under different physiological conditions [12–14]. Although cells possess various volume regulatory mechanisms to maintain the constancy of cell volume and also hydration status of the cell via the mechanisms largely by changing the permeability of various ions such as K^+^, Na^+^, H^+^, Cl^-^ and HCO_3_^-^, it has been noticed in many cell types that they remain in a slightly swollen or shrunken state for the duration of the aniso-osmotic exposure [12,13]. In comparison with mammals, teleost fishes face more problems of osmotic stress primarily owing to osmolarity changes in their external environment. Recently, presence of very efficient volume regulatory mechanisms have been demonstrated in hepatocytes of air-breathing catfish (*Clarias batrachus*) but the hepatocytes were found to remain in partly swollen or shrunken state as long as they were exposed to anisotonicity [15]. These minute changes of cell volume owing to anisotonicity have been reported to cause changes in carbohydrate metabolism [16–18], autophagic proteolysis [19] and protein synthesis [20] in this air-breathing catfish. Further, teleost fishes have been reported to face more problems of oxidative stress due to enhanced production of reactive oxygen species in presence of environmental contaminants, thus causing a threat to fishes [21–23]. Thus, fishes represent excellent models to identify and understand elements and mechanisms controlling the physiological and behavioral changes that occur in response to osmotic and oxidative stresses [22,24].

Aquatic systems are infested with various micro-organisms strictly aquatic bacteria, which are mainly of soil origin and carried into the water due to rain or to accidental introduction of natural or a direct consequence of human activity. Lipopolysaccharide (LPS) is a product of the cell walls of Gram-negative bacteria and one of the major causes of septic shock in humans, and in the activation of innate immune response [25,26] leading to production of proinflammatory cytokines, proteases, eicosanoids, and reactive oxygen and nitrogen species [27]. LPS induces many genes involved in the immune, infiammatory and acute phase responses. Among those genes, inducible nitric oxide synthase (iNOS) has been implicated in both protective and detrimental host responses to sepsis and endotoxemia [28–30]. Bacterial LPS, an endotoxin, has been reported to induce expression of inducible isoform of NOS in various cell types such as macrophages [31], vascular smooth muscle cells [32], astrocytes [33], and neurons [34,35]. An elevated production of NO, secondary to an increase in iNOS expression has been suggested to play an important role in the hyperemic effect of LPS in many vascular beds, including that of the brain [36,37]. Fishes are markedly tolerant to high doses of LPS compared with mammals (up to a 1000-fold) [38], and effects of LPS or Gram-negative bacterial infections become deleterious in fish after chronic infection, but are less severe after acute infection. Yao et al. [39] in their study showed *Edwardsiella ictaluri* administration resulted in different responses of NOS genes in channel catfish (*Ictalurus punctatus*).

The air-breathing singhi catfish (*Heteropneustes fossilis*), found predominantly in tropical Southeast Asia, is reported to be more resistant to various environmental challenges such as high environmental ammonia, hypoxic and desiccation stresses [40]. Further, they are reported to be euryhaline, inhabiting fresh and brackish waters as well as muddy marshes, thus facing wide variations of external osmolarity changes [41]. They frequently encounter the problem of osmotic challenges and environmentally-induced oxidative stress in its natural habitats especially in summer when the ponds and lakes dry up, thus compelling them to migrate inside the mud peat to avoid total dehydration, and during the monsoon season, when the water in the same habitat gets diluted. Further, the singhi catfish is also bacterially challenged in its natural habitats of dirty water bodies of ponds and lakes. Thus, the major objectives of the present study (i) was to investigate the possible induction of iNOS gene and more production of NO due to LPS (a bacterial endotoxin) treatment, and (ii) to determine the possible effects of osmotic stress, different stress hormones and oxidative stress on NO production in the perfused liver of iNOS-induced singhi catfish.

## Materials and Methods

### Animals

The singhi catfish (*H*. *fossilis*, weighing 80 ± 10 g body mass) were purchased from a single source that are bred and cultured in selected commercial ponds of Nilbagan Fish Seed Farm situated in Barpeta, Assam, India. Fishes were acclimatized in the laboratory approximately for 1 month at 28 ± 2°C with 12 h: 12 h light and dark photoperiods before experiments. No sex differentiation of the fish was done while performing these studies. Minced dry fish and rice bran (5% of body wt) were given as food every day, and the water, collected from a natural stream, was changed on alternate days. Food was withdrawn 24 h prior to experiments. The pH of natural stream water varied between 7.1–7.3 with oxygen, ammonia and nitrite contents ranging between 5.5–6.7 mg/L, 3.5–5.7 mg/L and 2.5–3.6 mg/L, respectively. At least 5 fishes were taken for each set of experiment along with 5 control fishes. Always 5 replicates were taken for each analysis for both treatments and controls. Fishes were anesthetized in neutralized 3-aminobenzoic acid ethyl ester (MS-222, 0.2 g/L) for 5 min before sacrificing by decapitation. The study was approved by the Institutional Animal Ethics Committee (IAEC) of North-Eastern Hill University, Shillong, India

For experiments with LPS, initially fishes were injected intraperitoneally with different doses of LPS (5, 10, 15, 20 and 25 mg/kg body wt) dissolved in 0.9% saline and the mortality rates of fish were recorded over a period of seven days with proper veterinary care. Fishes were continuously monitored about their behavior and movement in water after injection of LPS after every half an hour. No mortality of fish was recorded up to a dose of 15 mg LPS/kg body wt for a period of seven days without any sign of distress. However, fishes, injected with 20 and 25 mg LPS/kg body wt, started secreting mucous all along the body surface and were showing erratic swimming movement after 24 and 10 h, respectively, as an indication that the fishes were under distress and were about to die. The distressed fishes were immediately put in a heavy dose of anesthesia (MS-222, 0.5 g/L) to avoid the distress, which finally led to euthanasia. Further experiments with LPS were conducted at a dose of 12 mg LPS/kg body wt., where no pathological symptoms were noticed in any of the fishes. Proper veterinary care was also taken to all fishes injected with LPS. A group of control fish was injected intraperitoneally only with 0.9% saline solution. After 12 and 24 h of injection, blood was collected from the caudal vein with a heparinized syringe. Fishes were then sacrificed by decapitation after anesthetizing in MS-222 (0.2 g/L), and tissues such as liver, kidney, heart, gills, muscle and brain were dissected out and dropped into liquid nitrogen before storing at -80°C for further analyses.

### Estimation

The NO concentration in different tissues and plasma was determined spectrophotometrically at 540 nm following Griess reaction as described by Sessa et al. [42] after processing the tissue [43]. NO concentration in the effluent coming out from the perfused liver under different experimental conditions was also measured by the same method. A standard curve was prepared with sodium nitrite to calculate the NO concentration in different samples.

### iNOS assay

The iNOS activity was assayed spectrophotometrically [44] with certain modifications [45]. One unit of enzyme activity was defined as that amount of enzyme which catalyzed the formation of 1 μmole of citrulline per h at 30°C.

### Western blot analysis

Liver, kidney, heart, gills, muscle and brain tissues of both control and treated fish were homogenized in 20 mM Tris-HCl buffer (pH 7.4) containing 330 mM sucrose, 1 mM EDTA, 1 mM EGTA, 1 μM DTT, 1% Triton X-100 and a complete protease inhibitor cocktail (Roche, Germany), and sonicated for 30x4 s. The homogenate was centrifuged at 10,000 x g for 10 min at 4°C and supernatants were used for analysis of iNOS protein content by Western blot as described previously [45]. Immunodetection was carried out using an enhanced chemiluminescence kit (Bio-Rad, USA) according to the manufacturer’s directions. After exposure to X-ray film, bands were scanned and densitometric analysis was carried out in a Chemidoc (Bio-Rad).

### Immunocytochemistry and confocal laser scanning microscopy

Liver, kidney, heart, gills, muscle and brain tissues of both control and treated fish were excised quickly, washed in PBS (phosphate buffered saline), followed by soaking overnight in 4% paraformaldehyde, and then in 10% sucrose for 48 h at 4°C. Cryosections (10 μm) of fixed tissues were made at -24°C, spread on poly-l-lysine coated glass slides, air dried and stored at -20°C until staining. After fixation with pure methanol (-20°C, 5 min), sections were blocked with 1% bovine serum albumin (BSA, prepared in PBS) for 1 h. The iNOS antibody raised in mouse (1:20 dilution) was applied for 2 h in a wet chamber at room temperature. After washing with PBS (3x10 min), the slides were incubated for 2 h in Cy3-conjugated sheep anti-mouse IgG (cat # C 2181) (1:500, Sigma, St. Louis, USA) in a dark wet chamber. After final washing, the sections were covered with Vectashield mounting medium with 4',6-diamidino-2-phenylindole (DAPI) (Vector Laboratories, USA). Another set of slide was processed in the same way except incubation with primary antibodies, which served as negative controls. Immunostained sections were analyzed in a confocal laser microscope (Leica, TCS SP5, Germany). Cross-talk of fluorochromes was excluded by the use of the acousto optical tunable filter. The entire depth of a section was scanned in 1 μm steps. The resulting stacks of pictures were mounted as single projections.

### RNA extraction and cDNA synthesis

The total RNA was isolated from 50 mg each of liver, kidney, heart and brain tissues using TRI® Reagent (Sigma Chemicals, St. Louis, USA) [46]. The RNA solution was then further purified using the RNAase miniprotocol for RNA cleanup (Qiagen, Germany). Purified RNA was quantified using QIAxpert (Qiagen, Germany), diluted to 5 μg/μL and electrophoresed on 1% agarose gel stained with ethidium bromide to verify integrity. First strand cDNA was synthesized from 1 μg total RNA (DNase I-treated, Invitrogen, USA) in a total volume of 20 μL with Verso cDNA Synthesis kit (Thermo Scientific, USA) as per the standard protocol.

### Quantitative real-time PCR (qPCR)

Quantitative real-time PCR was performed in the StepOne™ Plus Real-Time PCR (Applied Biosystems, USA) with DyNAmo^TM^ Flash SYBR Green qPCR Kit (Thermo Fisher Scientific, USA). The reaction mixture of 10 μL each contained 5 μL of DyNAmo Color Flash SYBR Green, 0.5 μL of cDNA, 300 nM of each primer and nuclease free water. The PCR conditions were 95°C for 7 min, followed by 40 cycles of 95°C for 10 s and 60°C for 30 s. The qPCR was performed in triplicate and negative controls using no cDNA were run for each gene. Melting curve analysis was used to re-confirm amplification of only a single PCR product. The level of glyceraldehyde-3-phosphate dehydrogenase (GAPDH) was invariant between the control and treated fish validating its choice as an endogenous control. Fold changes of iNOS gene in treated fish compared to untreated controls were calculated using the modified delta-delta CT method [47].

The primer pairs were chosen from the published cDNA sequences of *Heteropneustes fossilis*: iNOS (Genbank accession no. GU322465) and GAPDH (GenBank accession no. LC076385). The primers for iNOS were: forward (5′-TG CAC ACA CTC AGC CCT ATG-3′) and reverse (5′-GAG AAG GAG GTT GCA CTG CT-3′) and for GAPDH the primers were: forward (5'-CA AGG CTG AAG GTG GCA AAC-3') and reverse (5'-GCA GAG GCC TTC TCA ATG GT-3'), designed with Primer Express Software 3.0 (Applied Biosystems, USA).

### Liver perfusion technique

Fishes were anesthetized in neutralized 3-aminobenzoic acid ethyl ester (MS222, 0.2 g/L) for 5 min before operation to perform the liver perfusion. Before performing the liver perfusion experiments, it was ensured that the fishes were totally paralyzed showing no physical movement. Isolated livers were perfused via the portal vein in a non-circulating manner with haemoglobin-free medium [48]. The isotonic medium (265 mOsmol/L, determined by freezing point depression method) contained 119 mM NaCl, 5 mM NaHCO_3_, 5.4 mM KCl, 0.35 mM Na_2_HPO_4_, 0.81 mM MgSO_4_, 0.44 mM KH_2_PO_4_ and 1.25 mM CaCl_2_ as a basic solution for perfusion. The perfusate was gassed with O_2_/CO_2_ (99:1, v/v), L-arginine at a concentration of 0.2 mM was also infused into the perfused liver and its pH was adjusted to 7.6. Livers were perfused at a flow rate of 4–5 ml/g liver/min and at a temperature of 28°C. The liver cells viability was continuously monitored by measuring the lactate dehydrogenase (LDH) release into the effluent, which never went beyond the level of 1.0–2.5 units/L in any of the experimental conditions. The effluent coming out of perfused liver was collected through a cannula catheterized at the superior vena cava for analysis of NO efflux. Livers were separated out from the body and weighed immediately after the perfusion. The NO fluxes were calculated with relation to the liver weight and the flow rate of perfusate, and were expressed as nmoles/g liver/min.

t-Butyl hydroperoxide (t-BOOH) and hydrogen peroxide (H_2_O_2_) (as causative agents for oxidative stress), adenosine, epinephrine and nor-epinephrine were also infused into the perfused liver through a precision pump (Orion, M361) along with the standard perfusion medium under different experimental conditions. Effluents were collected at 2 min intervals through a cannula inserted at the superior vena cava for the measurement of NO release from the perfused liver.

The hypotonic medium (-80 mOsmol/L) was prepared by removing the equivalent amount of NaCl, and the hypertonic medium (+80 mOsmol/L) was prepared by adding equivalent amount of NaCl to the standard perfusion medium as mentioned above. In another set of experiment, the anisotonicity of the medium was adjusted with mannitol in place of NaCl, i.e., the hypertonic medium was prepared by adding +80 mOsmol/L equivalence of mannitol to the standard perfusion medium, and the hypotonic medium was prepared by withdrawing +80 mOsmol/L equivalence of mannitol from the standard medium, where +80 mOsmol/L equivalence of NaCl was already replaced in the initial perfusion medium, thereby causing no change of NaCl concentration all through the experiment. In another set of experiment, glutamine plus glycine (2 mM each) were infused along with the isotonic medium in the perfused liver.

### Determination of water content in the perfused liver under different experimental conditions

The water content in the perfused liver was determined by oven drying method [16]. While measuring the effects of anisotonicity, oxidative stress, different metabolites or hormones as mentioned above on water content, livers were not perfused back with isotonic medium. The liver perfusion under different experimental conditions was stopped under treated condition, liver was weighed and kept immediately in an oven at 70°C for 24 h, which was found to be sufficient enough for complete drying of liver tissue. The difference between the wet and the dry weight of the perfused liver was taken as water content. The changes of water content in the perfused liver, under different experimental conditions as mentioned above, were calculated comparing with respective control experiments and were expressed as percentage changes of water content.

### Lactate dehydrogenase (LDH) assay

The LDH release, as a measure of cell damage under different experimental conditions, was assayed following the method of Vorhaben and Campbell [49] with certain modifications of substrate concentration [23].

### Chemicals

LPS (extracted from *Salmonella enterica* serotype typhimurium, L7261), oligonucleotide primers, enzymes, co-enzymes, substrates were procured from Sigma Chemicals (St. Louis, USA). The cDNA and qPCR kits were purchased from Thermo Scientific (USA). The iNOS (mouse monoclonal) (cat # Sc-7271) and secondary anti-mouse HRP- conjugated (cat # Sc-2354) were purchased from Santa Cruz Biotechnology (USA). Anti-mouse Cy3 conjugated antibody (cat # C2181) was purchased from Sigma Chemicals (St. Louis, USA). Other chemicals were of analytical grades and were obtained from local sources. MilliQ water was used in all preparations.

### Statistical analysis

The data collected from different replicates, were statistically analyzed and presented as mean ± S.E.M (*n*), where *n* equals the number of animals in each set of experiment. One way ANOVA test was performed to compare the experimental values with those of respective controls. When differences were indicated, Tukey’s post hoc test was used to determine homogeneous subsets. In all cases, α level of 5% (*P*<0.05) was selected to signify statistically significant differences.

## Results

### Tissue levels of NO in control and LPS-treated fish

**Fig 1:**
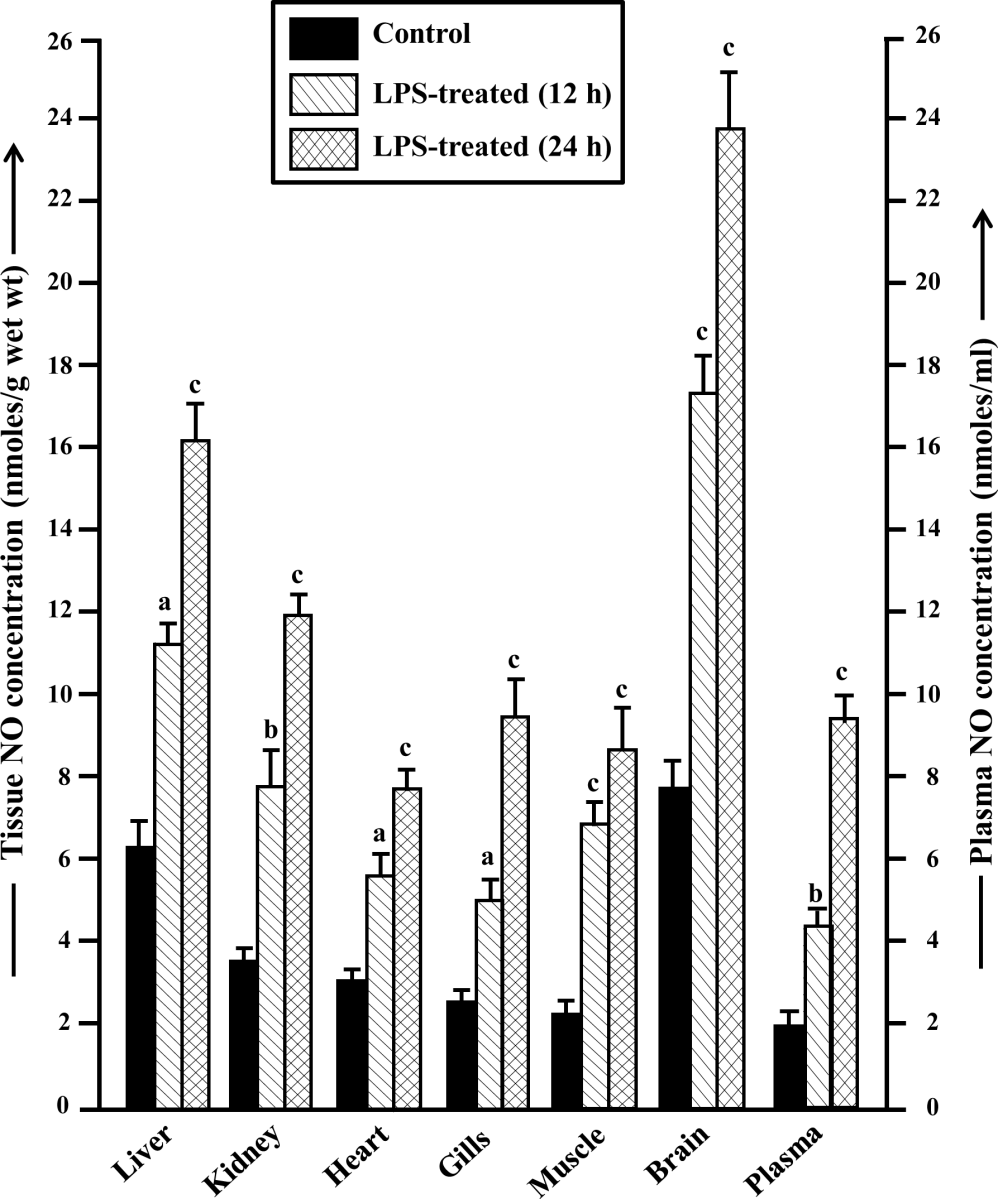
Tissue levels of NO in control and LPS-treated fish. Changes in concentration of NO in different tissues (nmoles/g wet wt) and in plasma (nmoles/mL) of *H*. *fossilis* following treatment with LPS. Values are plotted as mean ± SEM (n = 5). ^a,b,c^: *P* values significant at <0.05, <0.01 and <0.001 levels, respectively, compared to respective controls (Tukey’s post hoc test). LPS: Lipopolysaccharide.

Some amount of NO were found to be present in different tissues and also in plasma of control fish with a maximum level in brain, followed by liver, kidney, muscle, heart, gills and plasma in a decreasing order (Fig 1). However, after treatment with LPS for 12 and 24 h, the levels of NO in different tissues and in plasma increased significantly compared to control fish (Fig 1). After 12 h of LPS treatment, the concentration of NO increased maximally in muscle (2.81-fold), followed by kidney (2.29-fold), brain (2.27-fold), plasma (2.18-fold), heart (1.81-fold), liver (1.79-fold) and gills (1.78-fold). The concentration of NO enhanced further after 24 h of LPS treatment with a maximum increase in plasma (4.63-fold), followed by muscle (3.57-fold), kidney (3.48-fold), brain (3.13-fold), gills (2.64-fold), liver (2.58-fold) and heart (2.51-fold).

### Activity of iNOS in LPS-treated fish

**Fig 2:**
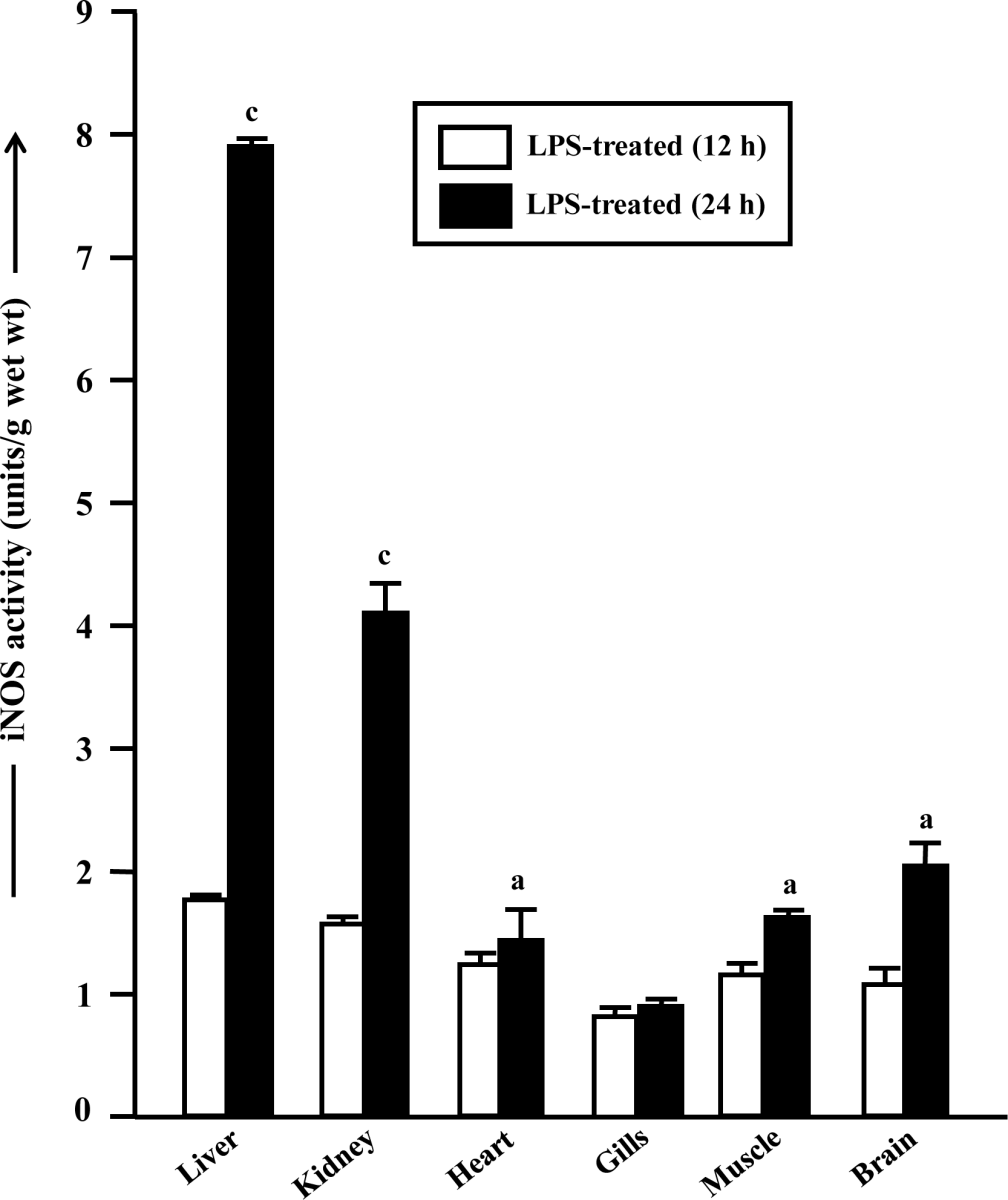
Activity of iNOS in LPS-treated fish. Changes in the activity of iNOS (units/g wet wt) in different tissues of *H*. *fossilis* after treatment with LPS. Values are plotted as mean ± SEM (n = 5). ^a,c^: *P* values significant at <0.05 and <0.001 levels, respectively, compared to respective controls (Tukey’s post hoc test).

The iNOS activity, which could not be detected in control fish by the assay method used, was able to detect in fish after 12 and 24 h of LPS injection (Fig 2). In 12 h LPS-treated fish, the iNOS activity was induced maximally in liver to a level of 1.74 units/g wet wt, followed by kidney (1.51), heart (1.24), muscle (1.16), brain (1.11) and gills (0.89 units/g wet wt). The iNOS activity was significantly induced further in all the mentioned tissues except in gills after 24 h of LPS injection by 4.54, 2.78, 1.16, 1.41 and 1.85-fold respectively, in liver, kidney, heart, muscle and brain, compared to 12 h LPS treated fish.

### Expression of iNOS protein in LPS-treated fish

**Fig 3:**
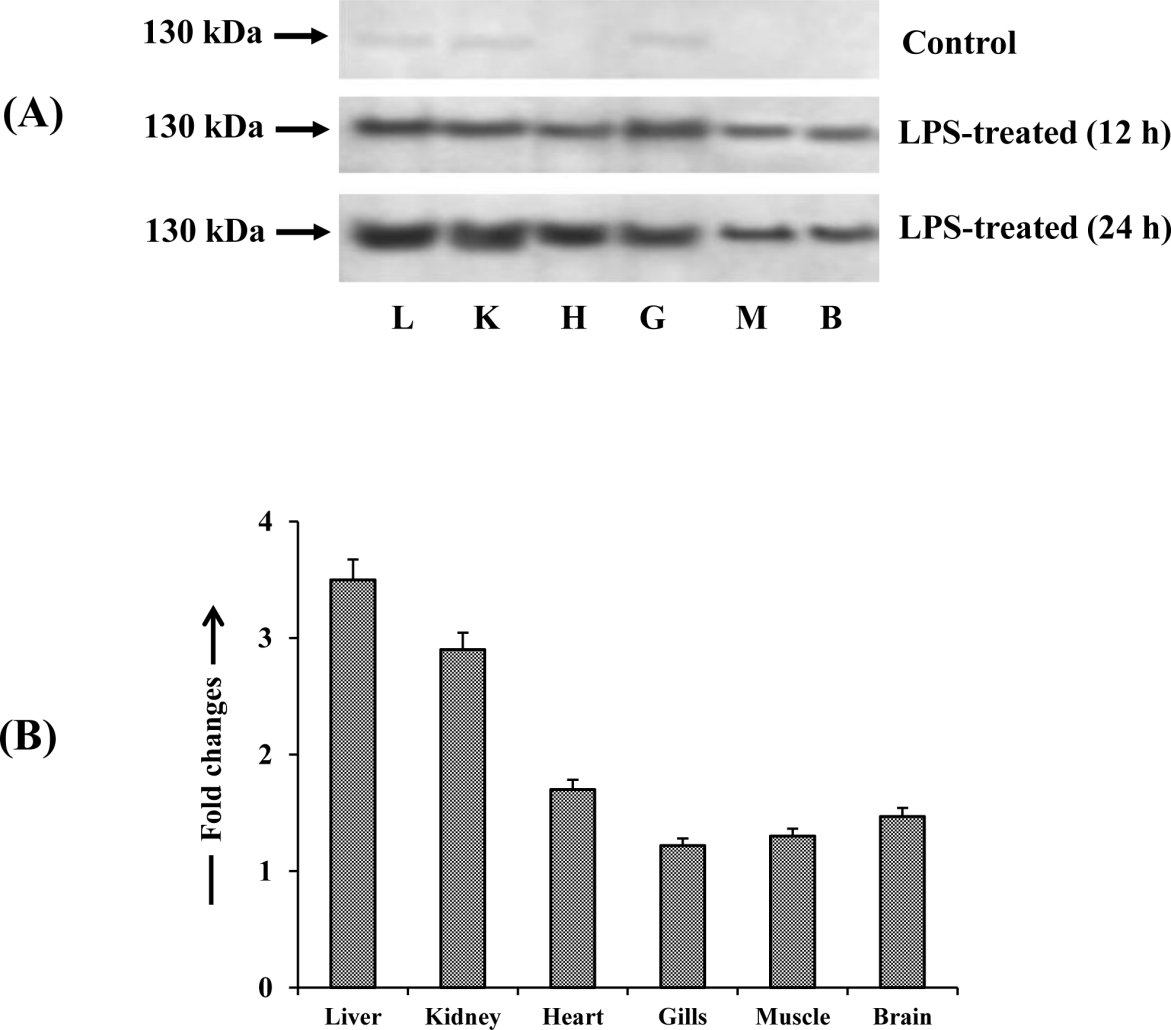
Expression pattern of iNOS enzyme protein. Western blot analysis showing the pattern of expression of iNOS protein in different tissues of control and LPS-treated *H*. *fossilis*. (A) Representative picture of five individual experiments is shown. (B) Densitometric analysis showing the fold increase of iNOS protein concentration in 24 h LPS- treated fish compared 12 h LPS-treated fish. Values are plotted as mean ± S.E.M. (n = 5). ^a,b,c^: *P* values significant at <0.05, <0.01 and <0.001 levels, respectively, compared to 12 h LPS-treated fish (Tukey’s post hoc test).

To characterize the distribution of iNOS isoform in different tissues of singhi catfish, the cross reactivity was confirmed by Western blotting technique using a specific antibody against iNOS (Fig 3). In 12 h LPS treated fish, an immunoreactive band of approximately 130 kDa, corresponding to the known iNOS molecular weight, was detected in liver, kidney, heart, gills, muscle and brain. The expression of iNOS protein in all the mentioned tissues increased further in 24 h LPS-treated fish as evidenced by the increase of band intensities by 1.2–3.5-fold compared to 12 h treated fish. However, in control fish no immunoreactive band was detected in most of the tissues except for a very thin band in liver, kidney and gills.

### iNOS immunolocalization in LPS-treated fish

**Fig 4:**
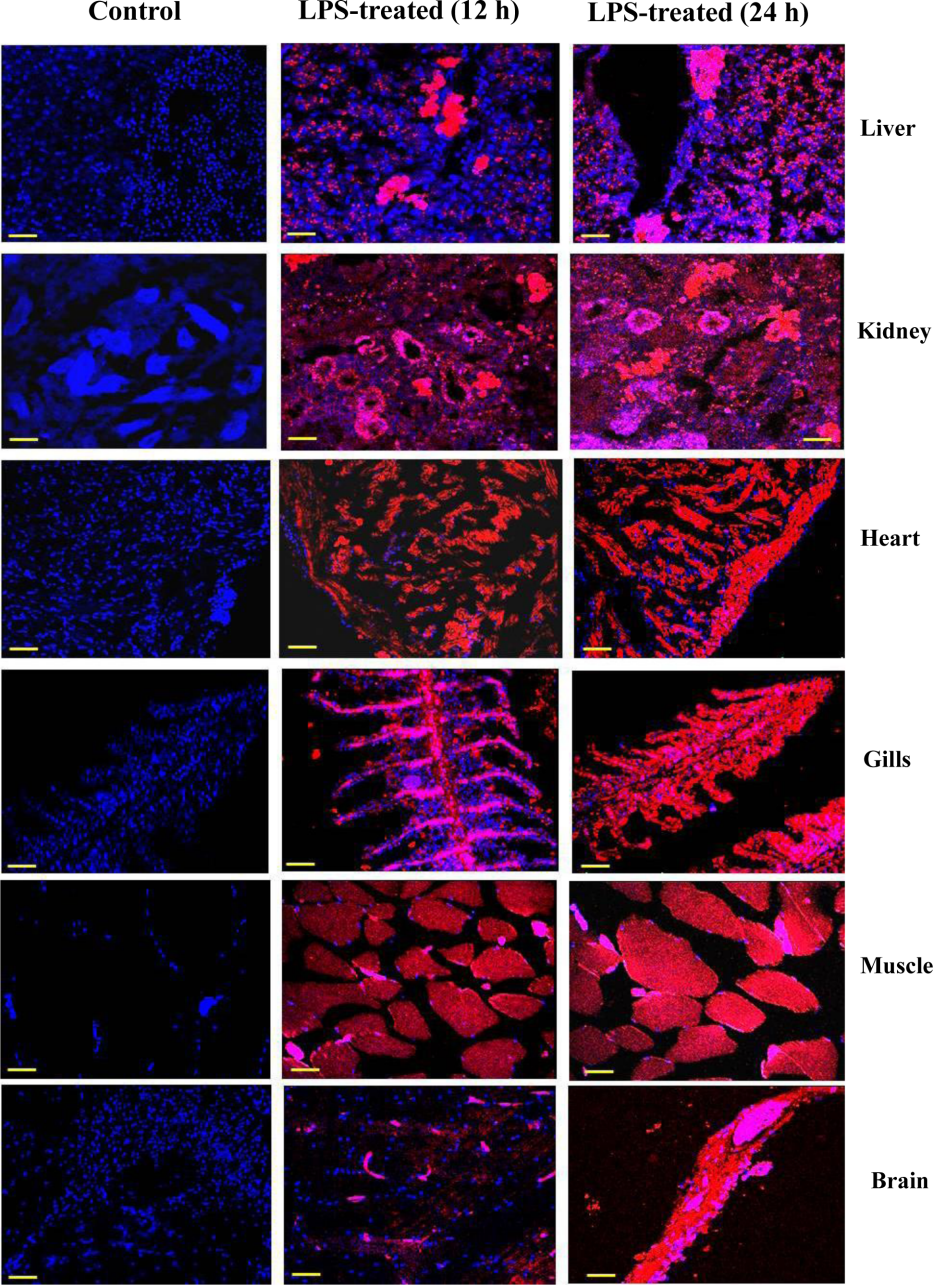
Zonal localization iNOS in LPS-treated fish. Immunocytochemical analysis showing the localized expression of iNOS in different tissues of *H*. *fossilis* after treatment with LPS. Representative pictures of five independent experiments are shown. Nucleus–blue; iNOS–red. Scale bar: 55 μm.

Confocal observations of expression and zonal localization of iNOS signal was made in different tissues of LPS-treated fish by immunodetection technique by using a monoclonal anti-iNOS specific antibody (Fig 4). Labeling specificity was confirmed by the absence of signal in parallel control sections treated without the primary antibody (data not shown). In liver of 12 h LPS-treated fish, very prominent signal for iNOS protein was observed mainly in the hepatic sinusoids and macrophages located near the blood vessels, which increased further after 24 h of LPS treatment. In kidney of 12 h LPS-treated fish, the iNOS protein was found to express in the glomerulus, proximal and distal tubules with its major confinement within the apical cytoplasm of the columnar epithelial and vascular endothelial cells. There was further increase in the expression of iNOS protein in 24 h LPS-treated tissue as evidenced by more prominent signal. In heart of LPS-treated fish, the iNOS signal was detected mainly in the atrium with absence of signal in the ventricular trabeculae. Therefore, in Fig 4, the iNOS signal is shown only in the atrial part of the heart. In 12 h LPS-treated fish heart, the iNOS was found to be localized at the level of endothelium endocardium (EE), which enhanced further after 24 h of LPS treatment. In gills, the iNOS signal was localized in the neuroepithelial cells in the interlamellar region of gill filaments and also in the cells of gill lamellae after 12 h of LPS treatment. The iNOS signal was also seen in nerve fibres, in the interlamellar vessels and the lamellar arterioles. Some traces of iNOS signal was also seen along the length of efferent edge and in the outer marginal channel, which consists of endothelial cells on its outer boundary. The immunolocalization was seen to increase further in gills of 24 h LPS-treated fish. In muscle the iNOS was found to express in the peripheral side of skeletal cell after 12 h of LPS treatment with further enhancement of iNOS protein expression in 24 h LPS-treated fish. In brain of 12 h LPS-treated fish, the iNOS protein was found to localize in the peripheral side of mesencephalon confining mainly to the glial cell which further increased after 24 h of LPS treatment. Whereas, in control fish liver, kidney, heart, gills, muscle and brain tissues no visible iNOS signal could be detected.

### Expression of iNOS mRNA in LPS-treated fish

**Fig 5:**
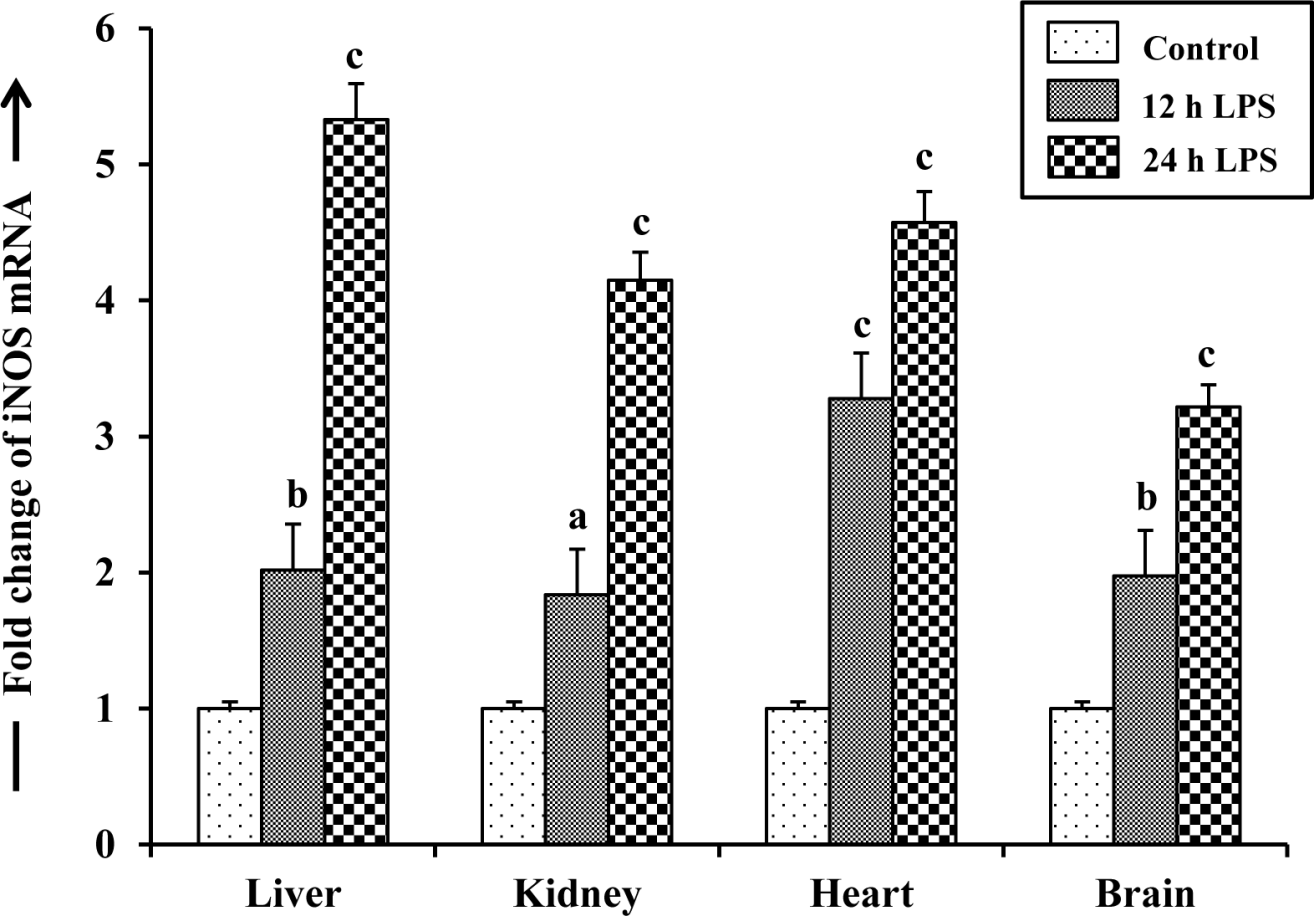
Expression of iNOS mRNA in LPS-treated fish. qPCR analysis showing fold changes of iNOS mRNA levels in different tissues of *H*. *fossilis* after treatment with LPS. Values are plotted as mean ± SEM (n = 3). L- liver, K- kidney, H- heart, B- brain. ^a,b,c^: *P* values significant at <0.05, <0.01 and <0.001 levels, respectively, compared to respective controls (Tukey’s post hoc test).

To further assess the possible induction of iNOS gene, the relative abundance of iNOS mRNA was determined by qPCR analysis with the total RNA isolated from liver, kidney, heart and brain tissues from 12 and 24 h LPS-treated fish in parallel with tissues from control fish (Fig 5). LPS treatment led to significant increase of mRNA levels in different tissues of singhi catfish after 12 h, followed by further increase after 24 h of LPS treatment. It increased maximally by 5.3-fold in liver, 4.6-fold in heart, 4.1-fold in kidney and 3.2-fold in brain after 24 h of treatment compared to control fish.

### Effects of anisotonicity on NO efflux from the perfused liver

#### (a) When the anisotonicity was maintained with NaCl

**Fig 6:**
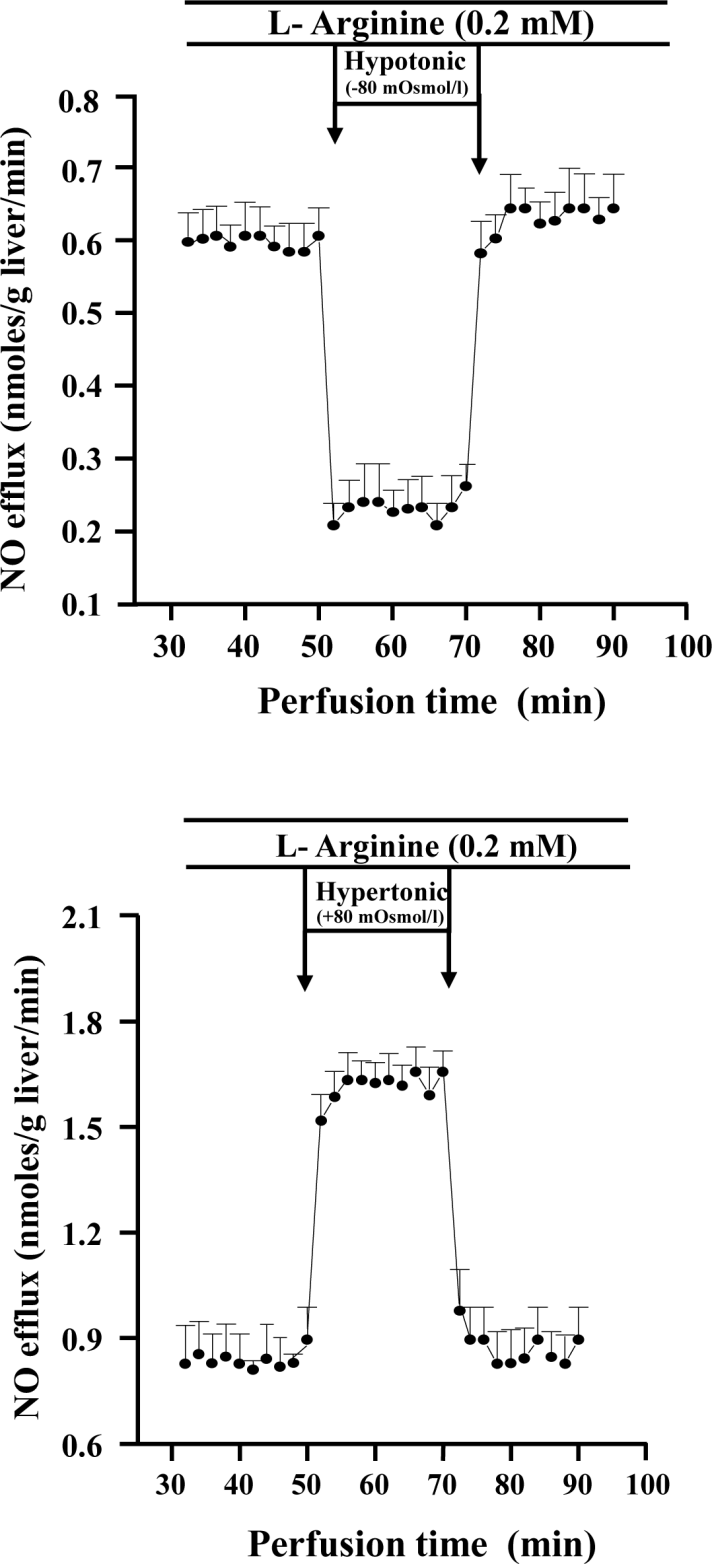
Effects of anisotonicity on NO efflux from the perfused liver. Effects of hypotonicity (-80 mOsmol/L) and hypertonicity (+80 mOsmol/L) (adjusted with NaCl) on NO efflux from the perfused liver of iNOS-induced *H*. *fossilis*.

**Fig 7:**
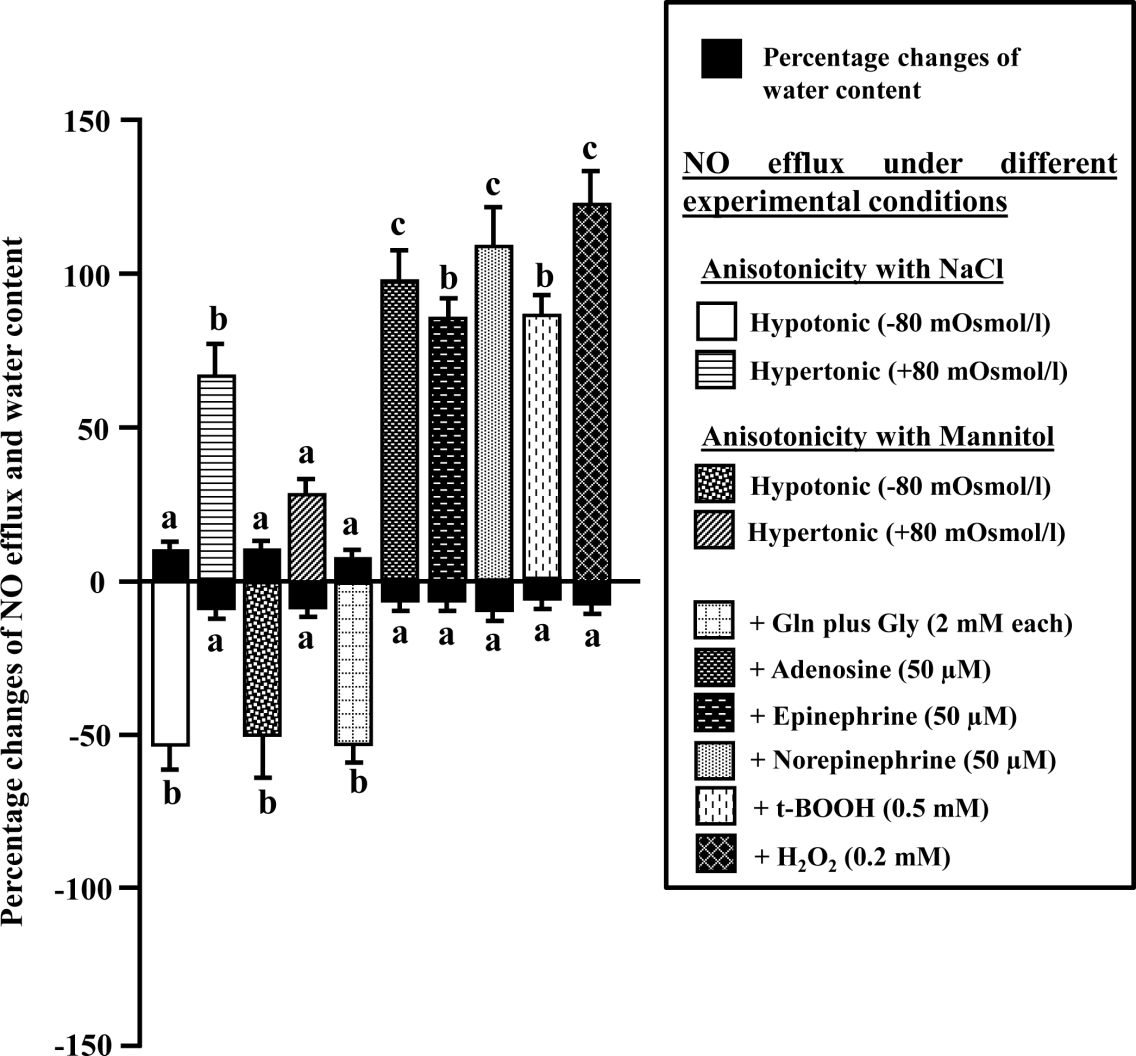
Influence of cell volume changes on NO efflux. Percentage changes of NO efflux from the perfused liver and the liver water content of iNOS-induced *H*. *fossilis* with respect to controls under different experimental conditions. Values are plotted as mean ± SEM (n = 5). ^a,b,c^: *P* values significant at <0.05, <0.01 and <0.001 levels, respectively, compared to respective controls (Tukey’s post hoc test).

As shown in Fig 6, when the iNOS-induced fish liver was perfused with hypotonic medium (-80 mOsmol/L), after initially perfusing with isotonic medium (265 mOsmol/L), the NO efflux decreased significantly from 0.59 ± 0.04 to 0.24 ± 0.07 nmoles/g liver/min (59%; *P*<0.01). The decrease of NO efflux persisted throughout the time period of hypotonic exposure, and returned almost to the basal level on restoration of isotonic medium. This decrease in NO efflux was accompanied by a significant increase (*P*<0.05) in the water content of the perfused liver by 12.5 ± 0.5% (Fig 7).

In contrast, when the iNOS-induced fish liver was perfused with hypertonic medium (+80 mOsmol/L), after initially perfusing with isotonic medium (265 mOsmol/L), the NO efflux increased significantly from 0.88 ± 0.07 to 1.64 ± 0.14 nmoles/g liver/min (86%; *P*<0.01) (Fig 6). The increase of NO efflux persisted throughout the time period of hypertonic exposure, and returned almost to the basal level on restoration of isotonic medium. This increase in NO efflux under hypertonic exposure was accompanied by a significant decrease (*P*<0.05) in the water content of the perfused liver by 10.9 ± 0.4% (Fig 7).

#### (b) When the anisotonicity was maintained with mannitol

When the anisotonicity of the medium was adjusted with mannitol instead of NaCl, similar effects of NO efflux from the perfused liver was observed (Fig 7). When the iNOS-induced fish liver was perfused with hypotonic medium (-80 mOsmol/L), after initially perfusing with isotonic medium (265 mOsmol/L), the NO efflux decreased significantly from 1.82 ± 0.17 to 0.91 ± 0.07 nmoles/g liver/min (50%; *P*<0.01). The decrease of NO efflux persisted throughout the time period of hypotonic exposure, and returned almost to the basal level on restoration of isotonic medium. This decrease in NO efflux under hypotonic exposure was accompanied by a significant increase (*P*< 0.05) in the water content of the perfused liver by 12.1 ± 0.6% (Fig 7).

In contrast, when the iNOS-induced fish liver was perfused with hypertonic medium (+80 mOsmol/L), after initially perfusing with isotonic medium (265 mOsmol/L), the NO efflux increased significantly from 1.68 ± 0.19 to 2.26 ± 0.22 nmoles/g liver/min (34%; *P*<0.05) (Fig 7). The increase of NO efflux persisted throughout the time period of hypertonic exposure, and returned almost to the basal level on restoration of isotonic medium. This increase in NO efflux under hypertonic exposure was accompanied by a significant decrease (*P*<0.05) in the water content of the perfused liver by 10.5 ± 0.5% (Fig 7).

### Effects of glutamine plus glycine on NO efflux

Addition of glutamine plus glycine (2 mM each) to the influent perfusate in isotonic perfusions, after initially perfusing with isotonic medium (265 mOsmol/L), led to a significant decrease of NO efflux from 1.31± 0.21 to 0.57 ± 0.03 nmoles/g liver/min (56%; *P*<0.01) (Fig 7). The extent of the decrease of NO efflux persisted throughout the time period of infusion of glutamine plus glycine, and returned almost to the basal level on withdrawal of these two amino acids from the isotonic medium. The decrease of NO efflux in presence of glutamine plus glycine was also accompanied by a significant increase (*P*<0.05) in water content of the perfused liver by 8.4 ± 0.4% (Fig 7).

### Effect of adenosine on NO efflux

When the iNOS-induced fish liver was infused with adenosine (50 μM), after initially perfusing with isotonic medium (265 mOsmol/L), the NO efflux increased transiently from 1.37 ± 0.14 to 3.08 ± 0.19, followed by a gradual decrease to a level of 2.63 ± 0.19 nmoles/g liver/min but remained higher by 92% (*P*<0.001) than the basal level (Fig 7). The extent of the increase of NO efflux persisted throughout the time period of exposure to adenosine, and returned almost to the basal level on withdrawal of adenosine from the perfusate. The increase in NO efflux in the presence of adenosine was accompanied by a significant decrease (*P*<0.05) in the water content of the perfused liver by 8.7 ± 0.4% (Fig 7).

### Effects of stress hormones on NO efflux

When the iNOS-induced fish liver was infused with epinephrine (50 μM), after initially perfusing with isotonic medium (265 mOsmol/L), the NO efflux increased transiently from 1.41 ± 0.99 to 3.78 ± 0.22 nmoles/g liver/min (168%). Following this, there was a gradual decrease of NO efflux to 2.42 ± 0.18 nmoles/g liver/min, but remained significantly higher than the basal level (71%; *P*<0.01), and was maintained at this level as long as the liver was infused with epinephrine (Fig 7). The NO efflux returned almost to the basal level when epinephrine was withdrawn from the isotonic medium. The increase in NO efflux in the presence of epinephrine was accompanied by a significant decrease (*P*<0.05) in water content of the perfused liver by 8.8 ± 0.4% (Fig 7).

Similarly, when the iNOS-induced fish liver was infused with norepinephrine (50 μM), after initially perfusing with isotonic medium (265 mOsmol/L), the NO efflux increased transiently from 1.58 ± 0.19 to 4.39 ± 0.51 nmoles/g liver/min (178%). Following this, there was a gradual decrease of NO efflux to 3.47 ± 0.25 nmoles/g liver/min, but remained significantly higher than the basal level (119%; *P*<0.001), and was maintained at this level as long as the liver was infused with norepinephrine (Fig 7). The NO efflux returned almost to the basal level when norepinephrine was withdrawn. The increase in NO efflux in the presence of norepinephrine was accompanied by a significant decrease (*P*<0.05) in the water content of the perfused liver by 9.4 ± 0.6% (Fig 7).

### Effect of oxidative stress on NO efflux

The oxidative stress was caused by infusing t-BOOH and H_2_O_2_ to the perfused liver. When the iNOS-induced fish liver was subjected to oxidative stress, by infusing of t-BOOH (0.5 mM), after initially perfusing with isotonic medium (265 mOsmol/L), the NO efflux increased transiently from 0.71 ± 0.02 to 2.13 ± 0.19 nmoles/g liver/min (200%). Following this, there was a gradual decrease of NO efflux to 1.33 nmoles/g liver/min, but remained significantly higher than the basal level (87%; *P*<0.01), and was maintained at this level as long as the liver was infused with t-BOOH (Fig 7). The NO efflux returned almost to the basal level when t-BOOH was withdrawn. The increase in NO efflux in the presence of t-BOOH was accompanied by a significant decrease (*P*<0.05) in the water content of the perfused liver by 9.4 ± 0.5% (Fig 7).

Similarly, when the iNOS-induced fish liver was subjected to oxidative stress by infusing of H_2_O_2_ (0.2 mM), after initially perfusing with isotonic medium (265 mOsmol/L), the efflux of NO increased transiently from 0.99 ± 0.19 to 3.78 ± 0.13 nmoles/g liver/min (281%), followed by a gradual decrease of NO efflux to 2.20 ± 0.27 nmoles/g liver/min, but remained significantly higher than the basal level (122%; *P*<0.001), and was maintained at this level as long as H_2_O_2_ was infused into the liver (Fig 7). The NO efflux returned almost to the basal level when H_2_O_2_ was withdrawn. The increase in NO efflux in the presence of H_2_O_2_ was accompanied by a significant decrease (P<0.05) in water content of the perfused liver by 8.2 ± 0.3% (Fig 7).

## Discussion

Fishes are constantly exposed to stressful conditions in their natural habitats and hence, they respond to stressors by opening up a variety of stress-sensitive pathways [50–53]. These stressors can upset delicate balances in cellular metabolic responses, generate reactive oxygen species (ROS), and lead to oxidative stress and cellular damages [54]. In addition to reports on various biochemical adaptations related to nitrogen metabolism in singhi catfish under environmental stresses [40], the role of NO has been emphasized in this fish under ammonia stress during exposure to high external ammonia and during desiccation stress by our group [45,55]. Further, in the present investigation, it was observed that the intraperitoneal injection of LPS, the bacterial endotoxin, led to induction of iNOS gene leading to enhanced activity of iNOS enzyme as a consequence of more abundance of iNOS protein, and subsequently more synthesis and accumulation of NO in different tissues of singhi catfish. However, the physiological implication of LPS mediated iNOS gene expression in this catfish is yet to be established, similar to the line as suggested in some other fish species such as in brown trout [56], European eel [57], rainbow trout [58] and several other teleosts [59–61]. In singhi catfish, the iNOS activity could be detected in liver, kidney, heart, gills, muscle and brain tissues after LPS treatment with maximum activity after 24 h, which was further confirmed by Western blotting using a specific iNOS antiserum. A distinct band for iNOS was seen in all the tissues after 12 h of LPS treatment with further increase of band intensities after 24 h of treatment.

Immunocytochemical analysis of iNOS protein indicated that LPS caused zonal specific expression of iNOS in different tissues rather than expressing uniformly all through the organs. In liver, the expression of iNOS was more predominant in sinusoidal cells surrounding the blood vessels, which probably acts in vasodilation in this air-breathing catfish under pathological condition. Production of NO in kidney, due to expression of iNOS mainly in proximal and distal tubules might have helped in restoration of glomerular filtration and renal blood flow in singhi catfish in presence of endotoxin under pathophysiological conditions as suggested in rat kidney [62]. In heart, the more production of NO due to iNOS expression in endothelium endocardium of the atrial part of the heart probably helped in cardiovascular readjustments such as a drop in heart rate and also in maintaining blood pressure under pathological condition as suggested in bull frog [63] and in fish heart [4]. Very prominent expression of iNOS enzyme was seen in neuroepithelial cells of gill lamellae and gill filaments of singhi catfish after LPS treatment. Gills are constantly in contact with the external environment including that of various pathological bacterial contaminants, thus more expression of iNOS in gills probably serve as the first line of defence in this fish as suggested in channel catfish (*I*. *punctatus*) [39], and also probably to increase its ability to fight against the infectious diseases [64]. In brain, the iNOS enzyme protein was predominantly expressed in glial cells suggesting that production of NO in glial cells of singhi catfish brain might be playing an important role in neurotransmission and vasodilation under pathological condition [65]. The cause of expression of iNOS in peripheral side of skeletal cells of singhi catfish and production of more NO during LPS-treatment are not fully understood at this moment.

LPS treatment also resulted in enhanced expression of iNOS mRNA in liver, kidney, heart and brain tissues by about 4–5 fold within 24 h of treatment as evidenced by qPCR analysis. Thus, it is evident that the increase of iNOS activity and more abundance of iNOS enzyme protein, observed in LPS-treated fish, were mainly due to upregulation of iNOS gene, which probably associated with to encounter against the host immune response in different tissues as suggested in channel catfish (*I*. *punctatus*) [39].

More interestingly, the efflux of NO from the perfused liver of iNOS-induced singhi catfish was found to be influenced by changes of osmolarity of the perfusion medium. Perfusion of liver with hypotonic medium led to a significant decrease of NO efflux, which was associated with increase of hydration status of hepatic cells. Whereas, the liver perfused with hypertonic medium resulted to more efflux of NO, which was associated with the decrease of hydration status of hepatic cells. One might argue that changes in NaCl concentration, varied to adjust the osmolarity of the medium, might have affected NO efflux from the perfused liver rather than changing of osmolarity, since there are already reports on the changes of membrane potential in rat hepatocytes due to changes of NaCl concentration [66]. Therefore, in another set of experiment, when the anisotonicity was maintained with mannitol instead of NaCl (without changing the NaCl concentration all through the experiment), the same effects on NO efflux were observed. Thus, these osmo-sensitive changes of NO efflux appeared to be associated with the changes of cell volume/hydration status of the hepatocytes and not due to the changes of extracellular Na^+^ or Cl^-^ activity. Further, the changes of NO efflux from the liver due to cell volume changes under anisotonic conditions could also be associated with osmosensitive activation and inactivation of different L-arginine transport channels and also due to changes in the abundance of channel proteins as suggested in murine macrophages [67].

Similarly, the decrease and increase in NO efflux from the perfused liver of iNOS-induced singhi catfish, observed in presence of glutamine plus glycine and adenosine, respectively, in the perfusion medium, were probably associated with changes in hepatic cell volume, since addition glutamine plus glycine caused increase and adenosine caused decrease of hepatic cell volume. Thus, it is not only the osmo-sensitive changes of hepatic cell volume, but also certain metabolites-sensitive changes of hepatic cell volume could also influence the NO production. Metabolites-sensitive changes of cell volume have also been reported to cause various metabolic changes in the perfused liver of air-breathing walking catfish [17,18]. The increase of NO efflux by adenosine could also be attributed to increasing the production of cAMP due to modulation of adenylyl cyclase as a consequence of activation of adenosine receptor by adenosine [68], since cAMP is known to serve as a potent stimulator of iNOS expression and NO production in rat aortic smooth muscle cells [69] and human gingival epithelial cells [70]. Further, it is to be noted that adenosine levels are elevated in inflammatory lesions and can modulate the various functions of cells involved in inflammatory responses [71, 72]. Thus, it is reasonable to speculate that production of more NO by adenosine may serve to play an important role in promoting inflammatory responses in fish hepatocytes.

The production of NO under different stresses and its role in different stress-related reactions are well documented mostly in mammalian system [8]. Stress reactions involve the production and release of stress hormones such as glucocorticoids, catecholamines, prolactin, etc., which are the key metabolites of stress systems [73]. Thus, we also analyzed the effects of catecholamines such as epinephrine and norepinephrine on NO production in the perfused liver of iNOS-induced singhi catfish. Interestingly, infusion of both these hormones in iNOS-induced perfused liver caused a significant increase of NO efflux with a concomitant decrease in water content of the perfused liver. Thus, it may be assumed that increase in NO efflux was probably associated with decrease in hydration status of fish hepatocytes in presence of these catecholamines as seen in the case of hypertonic perfusion condition. Further, catecholamines are known to enhance the expression of cationic amino acid transporters involved in cellular uptake of L-arginine in activated murine macrophages [67]. Same could be true in singhi catfish, thus enhancing the uptake of L-arginine as a substrate for iNOS from the perfusate, thereby more production and release of NO from the perfused liver. Increased production of NO in presence of catecholamines may play an important role in modulating the inflammatory responses in fish liver as suggested in human monocytes [74]. Further, earlier reports have also indicated that NO efficiently suppresses the stress discharge of catecholamines from the adrenal gland [75] and also from pre-junctional membranes [76]. It is necessary to mention that during stress reactions, considerable release of stress hormones occur and the cells become exposed to activated free-radical oxidation, suppressed energy production, decreased synthesis of most proteins and protein structure denaturation [8]. In such a situation, the NO production, promoted by catecholamines, in turn can block the discharge of these stress hormones, and directly protect cells and organs from stress damages [8]. Therefore, it may be hypothesized that NO production from fish liver may act through a feedback autoregulation for protection against stress-induced damages.

Further, there have been reports on decrease of oxidative stress in hepatic cells of air-breathing walking catfish (*C*. *batrachus*) due to cell swelling through the stimulation of hexose monophosphate (HMP) pathway and increase of oxidative stress due to shrinkage of liver cells mainly through the inhibition of HMP pathway [17]. Hence, it may be postulated that increased NO production and efflux by the perfused liver of singhi catfish was possibly associated with more imposition of oxidative stress under hypertonic condition. Whereas, decreased production and efflux of NO under hypotonic condition was probably associated with less imposition of oxidative stress. To confirm our hypothesis further, the iNOS-induced fish liver was perfused both with t-BOOH and H_2_O_2_ separately, both of which are frequently used as model compounds for studies on oxidative stress in hepatic cells [76]. Significant increase of NO efflux was observed from the perfused liver due to the infusion of both t-BOOH and H_2_O_2_, thus indicating that increased oxidative stress in hepatic cells resulted in increased production of NO. The increase in NO production in presence of t-BOOH and H_2_O_2_ was also accompanied by a significant decrease in water content of perfused liver. Hence, it may be contemplated that the increased NO production under oxidative stress was associated with decrease in hydration status of hepatocytes. Similar to our finding, t-BOOH and H_2_O_2_-induced decrease of hepatic cell volume was also reported earlier both in mammalian and fish hepatocytes [17,77,78].

In summary, from our results it is evident that more production and accumulation of NO that took place in different tissues of singhi catfish while treating the fish with LPS was mainly due to induction of iNOS gene and localized expression of iNOS enzyme in different tissues. It may be contemplated that the expression of iNOS enzyme and the production of NO are important machineries for enhancement in adaptability and survivality of this freshwater air-breathing singhi catfish when challenged with different bacterial contaminants in its natural habitats. Besides, it appears that changes in hydration status/cell volume of hepatocytes of singhi catfish, either due to changes of osmolarity or by addition of certain metabolites and hormones, are major determinants in regulating the production of NO in fish liver. However, the reasons of these osmosensitive changes of NO efflux from the liver of singhi catfish are not yet fully understood with the available data and awaits a detailed investigation to draw a definite conclusion. Further, more production and release of NO that was observed in the iNOS-induced perfused liver in presence of different stress hormones and under oxidative stress reflects the potential role of NOS enzyme system in various stress conditions as one of the biochemical adaptations and also its defensive role in cellular homeostasis. This is the first report of osmosensitive and oxidative stress-induced changes of NO production and efflux from the liver of any teleosts. Further, the level of expression of iNOS in this singhi catfish could also serve as an important indicator to determine the pathological status of the external environment. This could perhaps also be useful for better management of culturing this air-breathing singhi catfish both under natural and captive conditions.
